# Spark Plasma Sintering As a Solid-State Recycling Technique: The Case of Aluminum Alloy Scrap Consolidation

**DOI:** 10.3390/ma7085664

**Published:** 2014-08-06

**Authors:** Dimos Paraskevas, Kim Vanmeensel, Jef Vleugels, Wim Dewulf, Yelin Deng, Joost R. Duflou

**Affiliations:** 1 Department of Mechanical Engineering, University of Leuven–KU Leuven, Celestijnenlaan 300A, B-3001 Heverlee, Belgium; E-Mails: wim.dewulf@kuleuven.be (W.D.); yelin.deng@kuleuven.be (Y.D.); joost.duflou@kuleuven.be (J.R.D.); 2 Department of Metallurgy and Materials Engineering (MTM), University of Leuven–KU Leuven, Kasteelpark Arenberg 44, B-3001 Heverlee, Belgium; E-Mails: kim.vanmeensel@kuleuven.be (K.V.); jozef.vleugels@mtm.kuleuven.be (J.V.)

**Keywords:** spark plasma sintering (SPS), field activated/assisted sintering (FAST), solid-state recycling, aluminum (Al) alloys, chips consolidation

## Abstract

Recently, “meltless” recycling techniques have been presented for the light metals category, targeting both energy and material savings by bypassing the final recycling step of remelting. In this context, the use of spark plasma sintering (SPS) is proposed in this paper as a novel solid-state recycling technique. The objective is two-fold: (I) to prove the technical feasibility of this approach; and (II) to characterize the recycled samples. Aluminum (Al) alloy scrap was selected to demonstrate the SPS effectiveness in producing fully-dense samples. For this purpose, Al alloy scrap in the form of machining chips was cold pre-compacted and sintered bellow the solidus temperature at 490 °C, under elevated pressure of 200 MPa. The dynamic scrap compaction, combined with electric current-based joule heating, achieved partial fracture of the stable surface oxides, desorption of the entrapped gases and activated the metallic surfaces, resulting in efficient solid-state chip welding eliminating residual porosity. The microhardness, the texture, the mechanical properties, the microstructure and the density of the recycled specimens have been investigated. An X-ray computed tomography (CT) analysis confirmed the density measurements, revealing a void-less bulk material with homogeneously distributed intermetallic compounds and oxides. The oxide content of the chips incorporated within the recycled material slightly increases its elastic properties. Finally, a thermal distribution simulation of the process in different segments illustrates the improved energy efficiency of this approach.

## 1. Introduction

### 1.1. Energy and Material Efficiency Challenges in Aluminum (Al) Recycling

A trend towards resource-efficient manufacturing can be observed in recent years driven by public concerns for environmental protection and resource conservation, but also as a result of the prospective stricter policies on climate change [1]. A doubling of the resource demand can be expected by 2050, and in order to meet the carbon emissions target by 2050, the metal sector requires a 75% cut in emissions per unit output [2,3]. Thus, energy efficiency should be combined with material efficiency [2]. Al has been identified as one key base material. Its production was recently estimated to represent 3% of the total CO_2_ emissions of the industrial sector [3] or 1.1% of the world total CO_2_ equivalent. [4]. The production of Al from ore (primary production) is one of the most energy-intensive material production processes, consuming 173 MJ/kg [5] or 200 MJ/kg [6], depending on the applied technology and the energy mix used in the production. Secondary Al production from scrap requires much less energy. The theoretical energy to remelt and cast Al scrap is 1.14 MJ/kg [5]. However, despite considerable improvements in the energy efficiency of the melting furnaces, the overall energy consumption of the secondary production still can be as high as 7.7 MJ/kg [5] or even up to 20 MJ/kg [6], depending on the type of scrap, the furnace technology and the production energy mix.

Apart from the energy efficiency of the recycling phase, material efficiency is also a crucial factor. Globally, 41% of liquid Al becomes process scrap and, consequently, does not end up in the product, hence consuming a considerable amount of energy and resources in a no-added-value recycling loop [7]. Moreover, it is well known that a fraction of the liquid metal is lost due to oxidation during remelting. Oxidation losses highly depend on the scrap form. Light-gauge scrap, having a high surface area-to-volume ratio, typically ranging from 6 mm^2^/g to 7.7 mm^2^/g for turnings and chips [8], tends to float on the surface of the melt. This causes significant oxidation losses that can be as high as 16% [9] or even up to 25% [10]. These metal losses cannot be recovered, since the metal property is lost. The impact of material losses can only partially be compensated for by the oxidation heat, where this energy could contribute to the production energy input (for Al, this value is 31.05 MJ/kg). A scrap mass flow balance model presented by Boin and Bertam [11] for the reference year 2002 in the European Union (EU) shows that “turning scrap” (representing turnings, chips and cuttings) share a relatively large flow, around 18%, of the total new scrap mass in the EU (including imported scrap).

### 1.2. Solid-State Recycling Techniques

By avoiding remelting, significant amounts of both energy and metal can be saved. Thus, recently various solid-state recycling approaches have been developed and proposed targeting light metal consolidation at temperatures below the solidus temperature. Solid-state recycling techniques have been presented for Al [12,13,14,15,16,17], but also for Mg [18] and Ti [19] alloys. Severe plastic deformation (SPD) methods, such as equal channel angular pressing (ECAP), and hot extrusion are called upon to explore different deformation routes for solid-state scrap welding directly into bulk products or semi-products, avoiding the need for melting. The use of hot extrusion as a recycling method was introduced and patented by Stern [20] in 1945. Decades later, Tekkaya *et al.* [12] and Güley *et al.* [13], using a cold pre-compaction step to form Al alloy chips into a billet form, successfully hot extruded these chip-based billets directly into profiles, illustrating and optimizing this approach. They reported potential energy savings of nearly 90% compared to the conventional recycling route. Haase *et al.* [14] used complex extrusion dies, a porthole and an ECAP die to improve the chips’ welding quality, as well as the mechanical properties of the extrudates by introducing additional plastic strain into the material. Strengths and densities comparable with the base material can be achieved following this approach [12,13,14]. Paraskevas *et al.* [21] compared the environmental performance of the conventional recycling route of Al turnings by remelting and casting with the direct recycling route by hot extrusion. The environmental impact per mass of the chip-based profile is 57% lower than that of a cast billet-based profile, with an average value of 10% oxidation losses in the remelting process. Widerøe *et al.* [15] developed a direct screw extrusion method for shredded scrap, introducing rotational movement to the scrap compacting and extruding in one single step. Finally, Güley *et al.* [16] investigated the solid-state welding quality of the chips by using a criterion for the oxide layer breakage and an index for the welding quality. Plastic deformation should be large enough to crack the surface oxide layer of the chips in order to expose clean and non-oxidized metal surfaces together and allow the formation of adhesive metal bonds. The authors compared and confirmed their experimental results with the results from a finite element (FE) simulation.

A different approach was presented by Sherafat *et al.* [17], who recycled Al 7075 alloy chips with the use of commercial air atomized pure Al powder to fabricate a two-phase material of Al7075/Al. The mixture of chips and powder was cold compacted and hot extruded. Al powder acts as a binder and soft matrix and provides a better bonding for the chips. Solid-state recycling is applicable also for the rest of the light metals category. Wu *et al.* [18] recycled AZ31B magnesium alloy chips, while Luo et al. [19] recycled Ti machining chips into a fully-dense material, performing multiple passes from an ECAP die with the application of backpressure at elevated temperatures.

The majority of the above-mentioned solid-state recycling techniques are focusing on the hot extrusion process [12,13,14,16,17,18,20] or the modification of this process [15]. The production of elongated profiles is possible by this route. However, utilizing spark plasma sintering (SPS) technology allows the flexible production of near-net-shape parts or multiple parts in one cycle. SPS systems are already available at the industrial scale. Moreover, a field activated/assisted sintering (FAST) system that can efficiently produce near-net-shaped products with a cycle time below 1 min is under development [22]. These latest developments in the field can aid in the direction of the industrial implementation/scaling-up and valorization of the proposed approach.

### 1.3. SPS Description and Advantages

SPS is a pressure-assisted, pulsed electric current Joule-heated sintering method recently pioneered in the field of powder metallurgy (PM). SPS is also known as FAST, electric discharge compaction/consolidation (EDC), pulsed electric current sintering (PECS), plasma pressure compaction (P^2^C), pulse electric discharge process (PEDP), plasma activated sintering (PAS), electric field sintering, plasma pressure consolidation, pulse current pressure sintering (PCPS) and pulsed current hot pressing (PCHP). SPS is a non-conventional and versatile sintering technique for the rapid consolidation of metal or ceramic powders within a much shorter processing time and at lower temperatures compared to conventional PM processes [23,24,25,26]. The power consumption during SPS consolidation is about one-third to one-fifth of that of traditional techniques, including pressure-less sintering, hot pressing and hot isostatic pressing. A significant contribution to the development of electric current-assisted consolidation has been made by scientists from the USSR and post-soviet countries. A comprehensive review of these studies, which is outside the mainstream electronic databases, since many of them have been published in Russian, was compiled by Olevsky *et al.* [27].

During SPS, mechanical pressure is applied to compact powder in a die/punch set-up, while *in situ* generating very fast Joule heating by means of a high pulsed DC current flow. The success of the SPS method as a novel sintering method has been attributed to the role of the plasma that is generated between particles [24]. The action of this plasma to eliminate surface impurities is reported to be the reason for the observed enhanced sintering. Thus, the process inventors originally claimed that the pulses generated sparks and even plasma discharges between the particle contacts, which are why the process was named SPS and PAS [26]. It is frequently argued that the improved densification rates stem mostly from the use of DC pulses of high energy. Whether plasma is generated has not yet been directly confirmed in experiments. It has, however, been experimentally verified that the densification is enhanced by the use of DC pulses [28]. Aside from the influence of plasma generation, other obvious advantages of the SPS process include a fast heating rate and a more uniform heating condition. FE modeling work has been carried out by Vanmeensel *et al.* [29] to characterize the temperature distribution in the specimen/die/punch setup and its evolution during SPS; and by Giuntini *et al.* [30] in order to optimize the SPS tool design. One of the uses of SPS is to produce nanostructured Al alloys by utilizing flake and atomized powders [31] and coarse-grained high-density Al samples starting from pure Al powder [32].

### 1.4. Research Motivation and Objectives

The solid-state recycling techniques mentioned in Section 1.2 focus on light metal scrap consolidation by physical disruption and dispersion of the stable Al surface oxides that inhibit metal-metal bonding. The scrap consolidation is achieved by imposing significant amounts of plastic deformation into the material. Inspired by the potential of solid-state recycling in terms of energy and material efficiency (see Section 1.1), the use of SPS is proposed within this paper as a novel meltless recycling technique for light gauge Al scraps.

The SPS approach described within this work will investigate the viability of this technology as a solid-state scrap consolidation technique. To the authors’ knowledge, no sintered-based technique has been successfully applied in such a direction achieving a fully-dense, void-less material as an output. As the starting material for SPS-based solid-state recycling, Al alloy machining chips with characteristic dimensions of a few cm are used instead of fine powders that typically range from 50 μm to 150 μm, as used in conventional SPS applications. The research objectives of this paper are to prove the technical feasibility of this approach, as well as to characterize the recycled samples.

## 2. Materials

**Figure 1:**
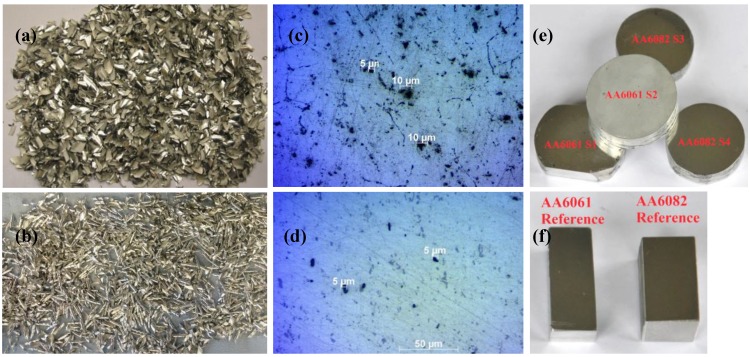
(**a**,**b**) The two different categories of chips that were used in this study (AA6061 (Al/1.0Mg/<0.6Cr/<0.6Cu/<0.6Si) and AA6082 (Al/1.0Si/0.9Mg/0.7Mn), respectively), produced by dry machining; (**c**,**d**) optical micrograph of the parent material (AA6061-T0 and AA6082-T651, respectively), as can be seen from the light optical microscope (LOM) (50× magnification); (**e**) the chip-based billets S1–S4 produced by means of spark plasma sintering (SPS);and (**f**) part of the parent materials that were used for the production of the chips.

Two types of different chip forms from two different alloys of the same age hardening alloy family, 6xxx, were used in this study. The first alloy was an AA6061 (Al/1.0Mg/<0.6Cr/<0.6Cu/<0.6Si) in T0 temper and the second was AA6082 (Al/1.0Si/0.9Mg/0.7Mn) in T-651 temper. Both chip fractions were generated by dry machining of the Al ingots, and two samples were produced from each chip batch. S1 and S2 produced from AA6061 chips, S3 and S4 from AA60823 chips. Figure 1 presents the two different chip forms, the microstructure of the initial cast material as can be seen from the light optical microscope (LOM) and the final recycled samples, along with their parent material.

The average size of the chips was 6 mm × 4 mm and 20 mm × 3 mm for the AA6061 and AA6082 batches, respectively. The machined chips produced from the AA6061 alloy had higher initial micro-porosity with observed pores ranging from 5 μm to 20 μm, compared to the chips produced from the AA6082 alloy with significantly less pores ranging from 5 μm to 10 μm. This can be explained, since wrought alloys are characterized by low “castability”. The AA6061-T0 alloy was received as casted ingots, in contrast with the AA6082-T651 alloy that was further rolled into plates, excluding residual porosity. The micro-porosity level of the parent material for each batch can be seen in Figure 1b,f. The measured density of the parent/reference alloys also confirms this fact, with the AA6061 ingot having a slightly lower density compared to the AA6082 (see Table 1).

**Table 1:** Density measurements of the recycled samples.

Sample	Measured density (g/cm^3^)	Oxide content (%)
S1–ΑΑ6061	2.807	8.48
S2–ΑΑ6061	2.806	8.49
Al6061-T0 reference	2.686	-
S3–ΑΑ6082	2.83	10.42
S4–ΑΑ6082	2.832	10.52
AA6082 T-651 reference	2.701	-

## 3. Experimental Section

### 3.1. Process and Tool Set-Up

The scrap metal chips were initially pre-compacted at room temperature in order to efficiently fill the SPS die. Based on the pre-compacted billet mass, the final billet size can be calculated. The SPS equipment type FAST, HPD 25/1, developed by FCT Systeme, Rauenstein, Germany, was used. The equipment consists of a 250-kN hydraulic press, a power supply system, a vacuum/gas chamber and a fully-automated thermal and hydraulic process controller capable of exerting uniaxial pressure and of applying pulsed or constant DC current.

**Figure 2:**
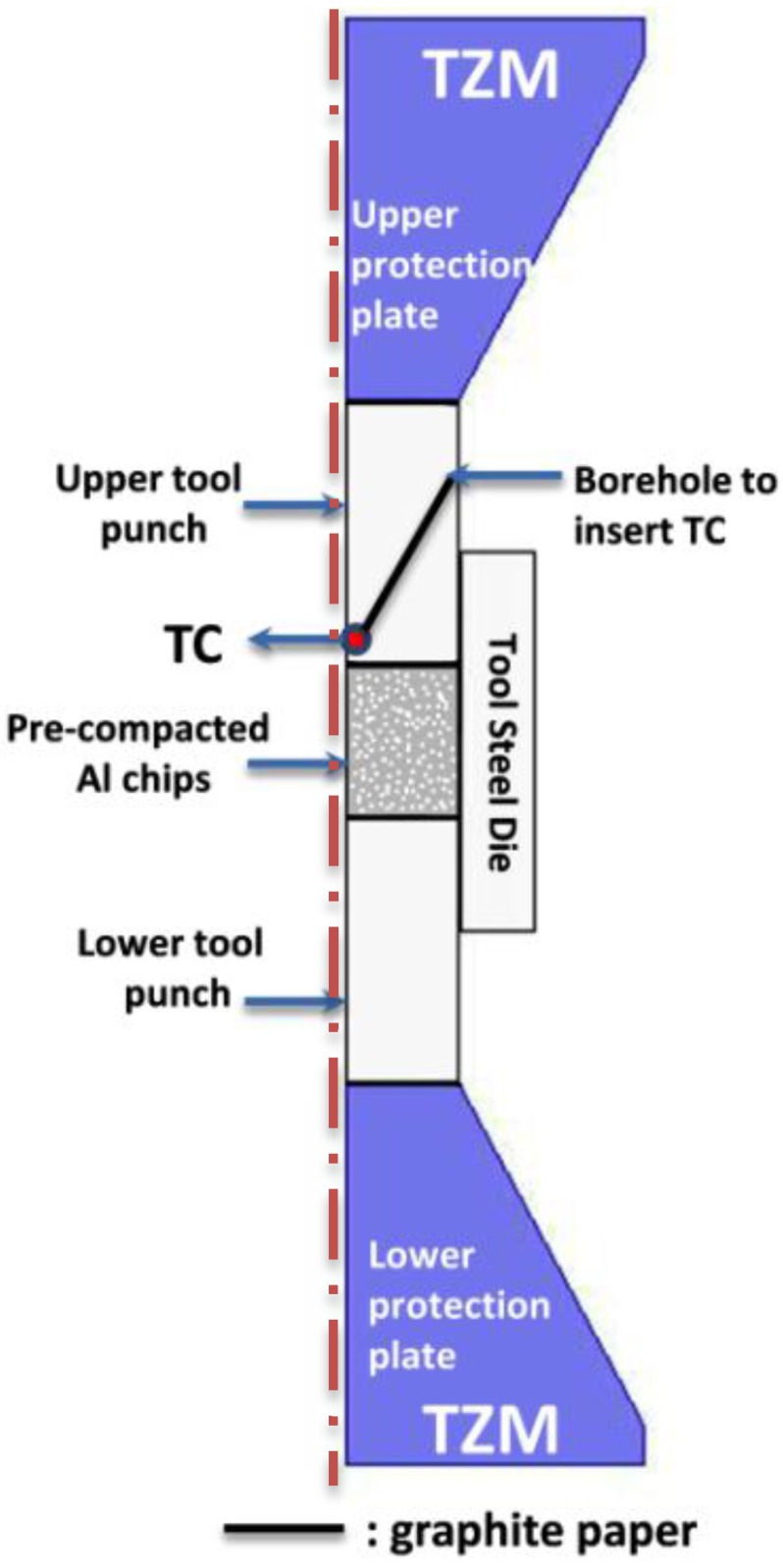
SPS tool set-up. TZM: molybdenum-titanium-zirconium; and *TC*: thermocouple.

A schematic cross-section of the die/pre-compacted sample/punch tool system is given in Figure 2. Steel dies and punches (Uddeholm Grade QRO 90, Hagfors, Sweden) and a 0.35 mm-thick graphite paper (Carbone Lorraine Grade N998, Courbevoie, France) at the punch/die and die/Al interfaces were used. Graphite paper was also used in between the punches and the metal to facilitate resistance heating and to avoid sticking of the compacted material to the punch and die. The process temperature was recorded by means of a flexible thermocouple (*TC*), which was positioned through a borehole at the bottom center of the upper cylindrical punch close to the sample (see Figure 2). A molybdenum-titanium-zirconium (TZM) alloy, well suited for high-strength and high-temperature applications, was used for the conical protection plates. A comprehensive description of the tooling materials used in the electric current-assisted consolidation set-ups can be found in Olevsky *et al.* [27].

### 3.2. Sintering Cycle and Precipitation Hardening

**Figure 3:**
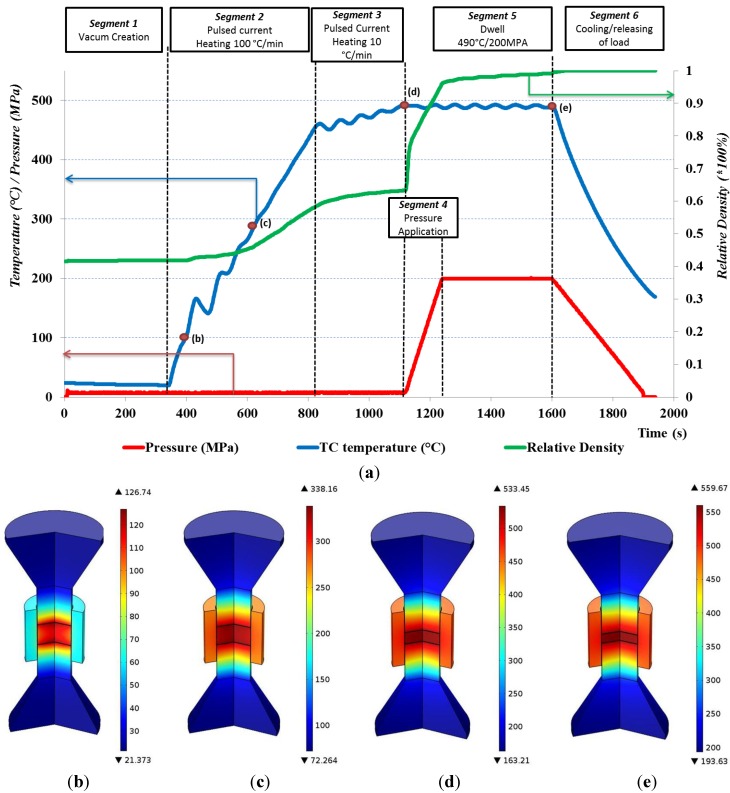
(**a**) The different segments of the sintering cycle. *TC* temperature, pressure and relative density (*RD*) *vs.* time; (**b**–**e**) thermal distribution simulation at different densification levels: (**b**) *TC* = 100 °C, *RD* = 41.5%; (**c**) *TC* = 293 °C, *RD* = 46.5%; (**d**) *TC* = 490 °C, *RD* = 77.4%; and (**e**) *TC* = 490 °C, *RD* = 100%.

The sintering cycle consists of the creation of vacuum in the surrounding vessel, the application of pulsed current until a temperature of 490 °C is reached, the application of mechanical load, dwell time during which the pulsed current is used to maintain the sintering temperature under maximum mechanical load and a cooling step, with subsequent release of the mechanical load. Throughout all of our experiments, a 10:5-ms pulse:pause pattern was applied. In Figure 3a, the used sintering cycle with the different Segments 1–6 and the process parameters are presented. The *TC* temperature, mechanical pressure and relative specimen density are plotted *versus* time for the different segments. During the process, Joule heating takes place in all electrically conductive components of the tool set-up. The amount of locally generated Joule heat depends on the local current density that is directly related to the electrical conductivity of the different tool parts. Heat is transferred by conduction and lost by radiation from the outer steel die and punch surfaces, especially at high temperatures, and by conduction of heat towards the TZM plates, which are in close contact with the water-cooled electrodes. Upon completion of the sintering cycle and after cooling down to 200 °C, the furnace vessel is filled with argon gas, the tool set-up is dismantled and the adhering graphite paper is removed from the specimens. The process time can be seen in the *x*-axis of Figure 3a. The pressure application segment is around 2 min, and the dwell segment is around 6 min.

Moreover, Figure 3b–e shows the results of a process thermal distribution simulation at different densification levels, as indicated in Figure 3a. A description of the underlying FE simulation model can be found in Vanmeensel *et al.* [29]. Densification is considered as complete when the shrinkage rate reaches zero at the beginning of Segment 6.

Solution treatment of the samples was partially performed during the sintering cycle at Segments 3–5. The thermal distribution simulation (Figure 3d) shows a temperature difference between the location of the *TC* in the punch (490 °C) and the center of the Al scrap metal compact that experienced a temperature above 520 °C. After quenching at room temperature in air, the samples were left to naturally age, after which hardness and density measurements were performed. Still, the material remained in an unstable state (W temper), and thus, artificial aging was used to supplement natural aging and to relieve possible internal stresses introduced due to the microplastic deformation during the dynamic scrap compaction step. Artificial or accelerated aging is also used in industrial practice after processing the 6xxx alloys (e.g., after hot profile extrusion). The artificial aging was performed in an air furnace at 175 °C for 4 h based on the industrial standards.

### 3.3. Material Characterization Measurements

After the typical metallographic preparation (grinding and polishing) of the samples’ surfaces, the following characterization measurements were performed.

#### 3.3.1. Microstructural Characterization

The microstructure and the chemical analysis of the top and cross-section surface of all samples studied were studied by scanning electron microscopy (SEM, Phillips XL-30 FEG and FEI Nova NanoSEM, Eindhoven, The Netherlands) with a backscattered electron detector and by LOM. The top surface of Sample S2 was chemically etched with the Barker agent in order to obtain better quality LOM micrographs.

#### 3.3.2. Hardness and Density Measurements

The Vickers hardness (HV_0.3kg_) was measured on the polished surfaces and cross-sections of all of the densified samples. For each sample, the average hardness value and concomitant standard deviation of five randomly selected Vickers indentations will be reported. The Vickers microhardness was determined using a hardness tester (model FV-700, Future-Tech Corp., Tokyo, Japan) with a 0.3-kg load and 10-s holding time.

The densities of the sintered samples were measured by Archimedes’ immersion method (BP210S Balance, Sartorius AG, Goettingen, Germany). After grinding and polishing the samples’ outer surfaces, the sample weight was measured in air (*m*_air_) and then in ethanol (*m*_eth_), and the density (*d*) then was calculated as:

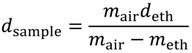
(1)

#### 3.3.3. X-ray Industrial Computed Tomography (CT)

CT is a non-destructive inspection technique based on differences in absorption/attenuation of X-rays through the material. The CT-technique provides a 3D data set of the sample. The attenuation of X-rays depends on the atomic number, density and thickness of the material and on the energy of the X-ray beam. The internal structure and a tomodensity analysis of a recycled sample were investigated through an industrial computed tomograph. With this technique, 2D, as well as 3D images and video fragments can be generated. Residual micro-porosity, cracks, material homogeneity, intermetallic inclusions and impurities can be detected using the non-destructive inspection capacity of the CT analysis. A Nikon XT H225 CT (Tokyo, Japan) was used for the described analysis. Sample S3 (AA6082) was mounted on polyurethane foams and then placed on the specimen plate in the CT device. The scans were configured at 145 kV and 165 μA. The obtained data have been analyzed using the VGStudio MAX 2.2.1 software package developed by Volume Graphics GMBH (Heidelberg, Germany).

#### 3.3.4. Impulse Excitation Technique (IET)

The IET technique was used to non-destructively determine the room temperature Young’s modulus and Poisson ratio of the different metal scrap samples after hot compaction. IET is a non-destructive technique that extracts a material’s elastic and damping properties based on the analysis of the vibration of a test sample after it was “impulse excited”. The resonance frequencies are characteristic for the specimen, as they are related to its stiffness, mass and geometry. The relationship between resonance frequency and Young’s modulus is accurately known for simple shapes, such us bars (reference material samples) and disks (recycled samples) [33,34], making IET a standard method to determine elastic moduli. The appropriate fundamental resonant frequencies, dimensions and mass of the specimens are used to calculate the dynamic Young’s modulus, dynamic shear modulus and Poison’s ratio according to the ASTM standards [33,35].

#### 3.3.5. Compression Testing

The mechanical behavior of the material was assessed by compression testing using an Instron Model 4467 Device (Norwood, MA, USA). For this reason, specimens with ϕ = 6 mm were cut by wire electrical discharge machining (EDM) out of the sintered compact. Prior to EDM, the sintered compacts were ground plan parallel. Compression tests were performed using a constant displacement control (0.2 mm/min) at room temperature. The corresponding ASTM standard was followed for the compression tests [34].

## 4. Results and Discussion

### 4.1. Microstructural Investigations

#### 4.1.1. Factors that Contribute to Oxide Layer Breakage and to Scrap Consolidation Process

**Figure 4:**
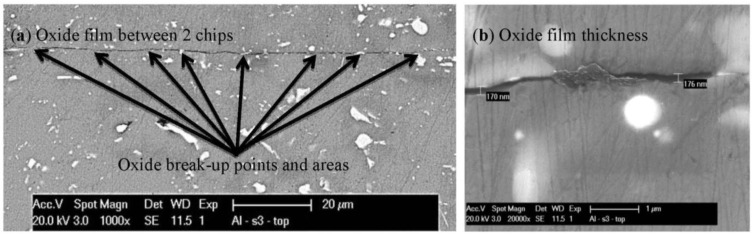
Scanning electron microscopy (SEM) micrographs of the top surface of the Sample S3: (**a**) oxide film between the boundaries of two chips and various oxide break-up points/areas; and (**b**) additional magnification revealing a welded and non-welded area with ~170-nm oxide film thickness.

**Figure 5:**
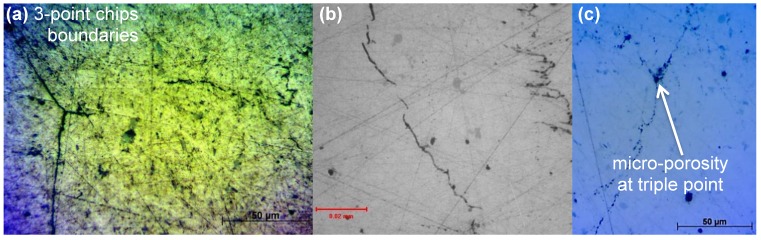
Oxide fracture as can be seen from LOM: (**a**) Sample S3: top surface, 50× magnification; (**b**) Sample S1: top surface, 50× magnification; and (**c**) Sample S4: top surface, 10× magnification.

Traditionally, Al products are considered very difficult to sinter and their properties deemed poor. This is because Al powder particle surfaces are always covered with a surface oxide film that cannot be broken and/or removed by heating. However, recent research has demonstrated that it is possible to improve solid-state sintering of Al powders or flakes with the use of SPS [31,32,36,37,38], even achieving highly dense samples [38]. In this study, SPS achieved sufficient solid-state chip welding by efficiently breaking the oxide surface layer of the chips, as can be seen from Figure 4 and Figure 5. Welded and non-welded areas are visible in all micrographs. The oxide film between the boundaries of two chips can be seen in the SEM micrographs of Figure 4. The regions with white color are the intermetallic compounds, and in Figure 4a, there are also visible points and areas where the oxide film is broken. An additional magnification of Figure 4a allows estimating the oxide film thickness, which is around 170 nm (Figure 4b), including potential air entrapped within the oxide layers.

It is argued that in the initial stage of the sintering process, the ON-OFF pulsed direct current generates a spark discharge between the particles [39,40], in this case between the Al chips. The gases existing in the sample can be ionized and transformed into plasma. However, experimental evidence for the spark discharge and/or plasma formation has not yet been unambiguously presented [41] and has also not been confirmed in this study.

Groza [23] attributed the enhanced densification during SPS to three possible mechanisms: (1) activation of the particle surface; (2) resistance to sintering; and (3) pressure application. Experimental evidence shows that particle surfaces are “cleaned” at an atomic scale, and direct grain-to-grain contact is observed in SPS-sintered specimens [42].

The authors believe that the interfering surface alumina films of the chips are pierced when a certain voltage level is achieved. The spark discharges and plasma generated promote the desorption of gases and breaking-up of the alumina layers on the surface of Al chips; hence, the purification and activation of the chips surfaces. Where a spark occurs, the surface oxide breaks-up, allowing metal-to-metal welding. This leads to favorable diffusion bonding, accelerating the consolidation process. Sparks are more likely to occur at inter-chip contacts at the initial stage of the compaction under low pressure and high current density (Segments 2–4). During compaction due to plastic deformation, the contact area increases with increasing compaction pressure, which makes melting and/or vaporization of entrapped gases less likely to occur.

**Figure 6:**
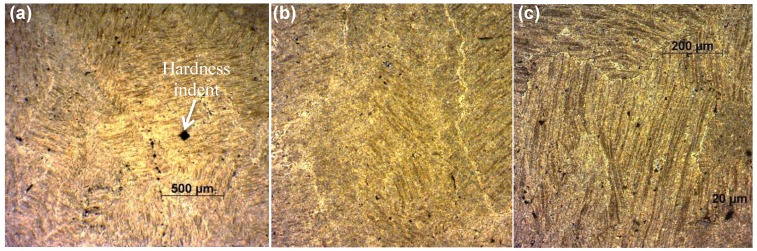
Solid-state chip welding as can be seen from LOM. Sample S2 top surface after chemical etching: (**a**) 4× magnification; and (**b**,**c**) 10× magnification.

Beyond the electrical discharge stage, the sintering parameters in SPS have a similar effect as in conventional pressure sintering, like hot pressing. After the chips’ oxide layers have been partially broken, diffusional processes and plastic flow are the main contributors to the densification in this stage. The highest temperatures achieved provide the highest diffusion rates and, thus, enhanced matter transport towards the metal-to-metal contact area. Thus, field application intensifies the sintering rate. In addition, the localized plastic flow is also enhanced by the combination of applied pressure and high temperatures. Figure 5 presents the microstructure of the samples as can been seen from an optical microscope and LOM. The chip boundaries are visible. The surface oxide layer of the chips (Figure 4) was efficiently fractured in many parts for all samples, allowing direct metal-to-metal contact. In some areas among the chips’ oxide interfaces, entrapped air/micro-porosity was visible. Generally apart from the original micro-pores that were identified in the parent material, ranging from 5 μm to 20 μm, no significant residual porosity was observed with LOM and SEM. The quality of the solid-state chip welding can be seen in Figure 6.

#### 4.1.2. Material Texture

**Figure 7:**
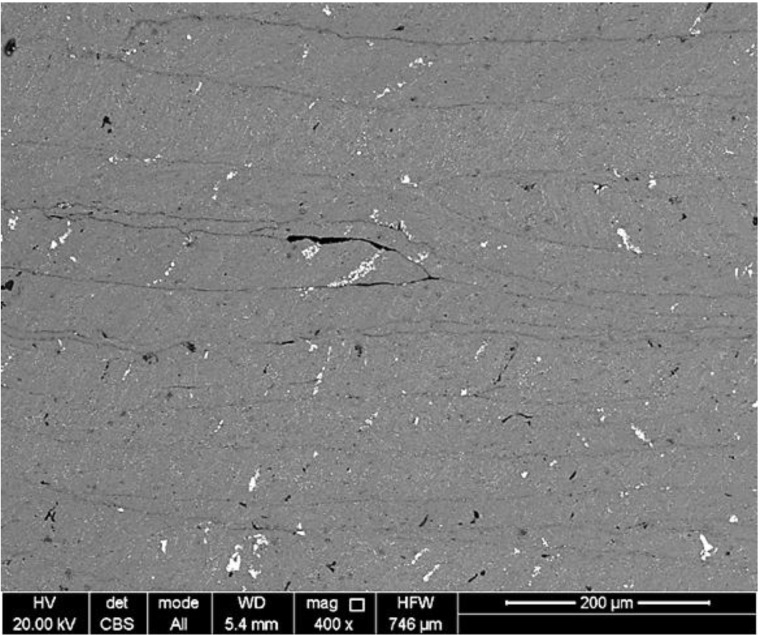
Cross-section SEM of S1.

At the cross-section of the samples, a lamellar topology can be observed. The chips are oriented orthogonal to the applied pressure axis. Figure 7 shows this lamellar texture at the cross-section of Sample S1 as observed by SEM. The chip boundaries are visible. A minor void of entreated air or micro-porosity among the chips can also be observed in this Figure 7. Generally, few similar voids were observed at the samples’ cross-sections. Due to this lamellar texture, the mechanical behavior of the samples was examined by means of the compression testing in both top and cross-sectional planes.

#### 4.1.3. Intermetallic and Grain Boundaries

**Figure 8:**
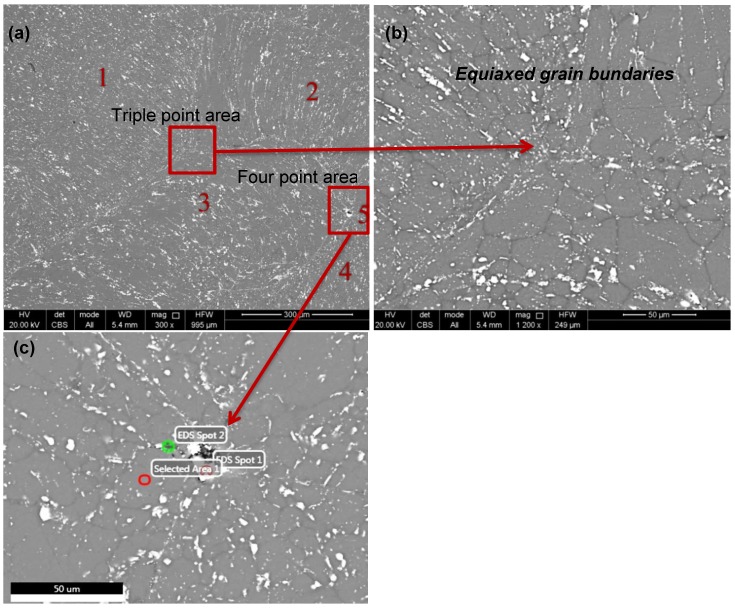
(**a**) Sample S4 top surface as can be seen from SEM/EDS; and (**b**,**c**) further magnification of the triple- and four-point area.

Figure 8 presents a typical microstructure of the top surface of the samples as can be seen by SEM with backscattered electron imaging of the chemical phase difference.

The white particles are intermetallic, and from their orientation (Figure 8a), different chips and their boundaries can be identified. Figure 8a is an overview of an area with five welded chips (mentioned with Numbers 1–5). A triple point where three chips are welded, as well as a possibly four-point area were further magnified in Figure 8b,c, respectively. Moreover, a qualitative and quantitative elemental analysis with energy-dispersive X-ray spectroscopy (EDS) in three EDS spots of Figure 8c is presented in Table 2. Copper intermetallic compounds in low concentration were detected in all EDS spots. Apart from copper, also Si, Mg and Fe intermetallic have been detected. The analysis of EDS Spot 1 reveals a high concentration of bismuth (89.5 wt%), a minor alloying element that is added to Al to assist in chip formation and to improve machinability. In Figure 8b,c, the grain boundaries are visible in the background. The grain boundaries range from 10 μm to 60 μm. The equiaxed grains are an indication of the recrystallization during the process. The analysis of EDS Spot 2 shows that alumina concentrates also in the grain boundaries (grey lines).

**Table 2:** Energy-dispersive X-ray spectroscopy (EDS) chemical analysis with weight%/(error%).

Peaks	Spot 1	Spot 2	Selected area
O K	3.83/(15.47)	9.91/(9.48)	-
Fe L	1.51/(62.38)	-	-
Al K	2.66/(9.9)	86.47/(2.38)	95.98/(1.9)
Bi M	89.52/(1.46)	-	-
Cu K	2.49/(14.42)	3.63/(9.16)	4.02/(6.8)

### 4.2. Densification, Oxide Content and Impurities

The density results obtained with the Archimedes method are presented in Table 1. All of the samples had higher density compared with the density of their parent Al alloys. This can be explained by the high oxide content of the chips. Fine-form scrap has a very high surface area-to-volume ratio, and thus, the oxide content is much higher than for solid samples (*d*_Al_2_O_3__ = 3.95 g/mm^3^, *d*_Al_ = 2.7 g/mm^3^). A typical specific surface value for the chips and turning scrap category can range from 6.0 mm^2^/g to 7.7 mm^2^/g [8]. Since the oxide film of the chips cannot be removed, it persists in the sintered product. Thus, assuming no voids and no foreign impurities in the material, an indirect estimation of the oxide volume fraction (%) can be calculated according to Equation 2. The oxide content of the samples depends: (I) on the form of the chips (surface area-to-volume ratio); and (II) the level of their oxidation. S1 and S2 that were produced by the AA6061 chips (Figure 1a) have ~8.5% oxide content, and S3 and S4 coming from the AA6082 chips (Figure 1b) have a higher content ~10.5% (see Table 1).


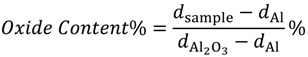
(2)

#### X-ray Industrial CT Analysis

**Figure 9:**
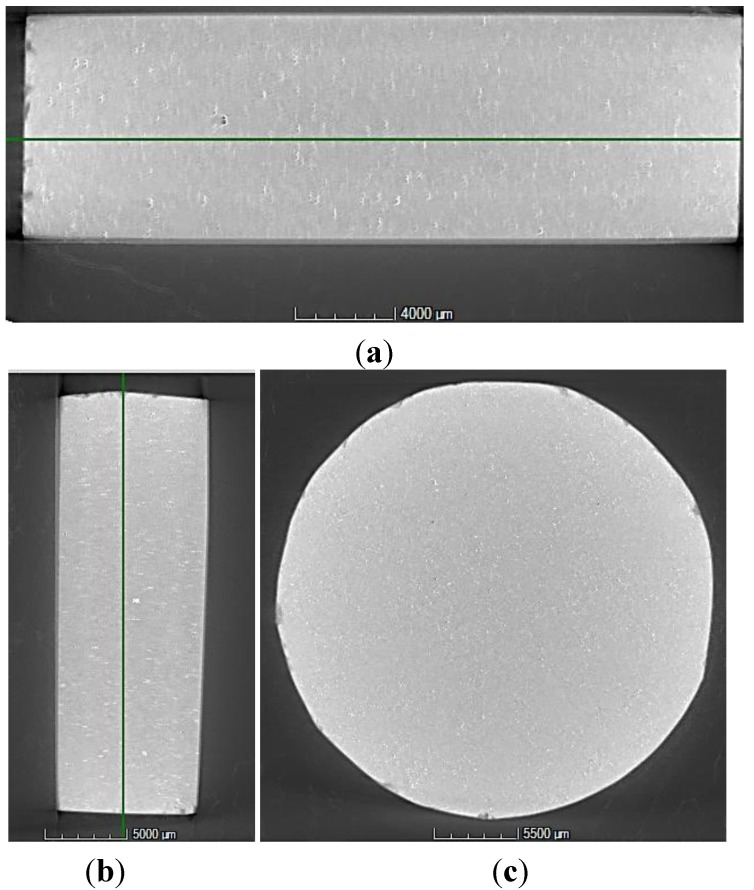
Computed tomography (CT) images according to three orthogonal planes for Sample S3: (**a**) *xy* plane; (**b**) *yz* plane; and (**c**) *xz* plane.

The produced SPS sample was CT scanned for internal structure evaluation. The derived 3D model of the specimen was analyzed slice per slice, obtaining the full picture of the sample. The whole set of cross-sectional frames was post-processed. The bulk area was packaged into video clips in the three planes (*xy*, *xz*, *yz*). Figure 9 provides a typical three-view image and a sample overview in which no pores can be identified. An in-depth analysis revealed that the CT measurement has a voxel size of 25.6 μm in the *X*, *Y* and *Z* direction. For visual detection, a size of at least three voxels is required. Assuming a spherical porosity would imply a threshold radius of 38.4 μm.

The CT findings confirm a sound solid-state welding capacity of the SPS process, since no porosity (with >40 μm radius) was detected within the global sample structure. In particular, the microscopic analysis highlights that the pore size of the parent material (pores of the chips) is mostly distributed in a range of 5–20 μm.

Thus, these micropores elude the CT imaging. The result couples to the density measurements in which fully-dense samples are acquired. Moreover, the CT images also reveal the presence of Al_2_O_3_. The brighter dots/areas with higher gray values, which are ubiquitously presented in the images of Figure 9a–c, are believed to be Al_2_O_3_ and intermetallic, thanks to their different absorption capacity for X-rays. It can be concluded that the Al_2_O_3_ is homogenously dispersed within the sample. Finally, the white edge in the images is beam hardening, which is a well-known CT artifact.

**Figure 10:**
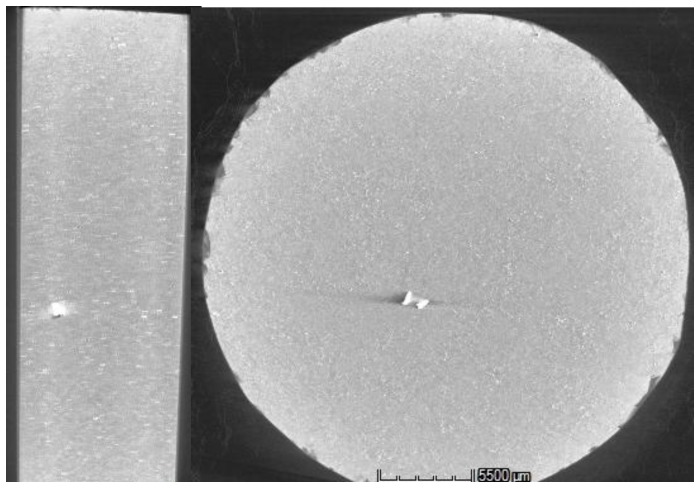
CT images revealing an impurity within the material.

An area with notably elevated gray values was observed near a sizable pore, which is further shown in Figure 10. This indicates the presence of an impurity, possibly iron. The impurity resists the welding process between Al chips, which creates a dislocation and, subsequently, results in the pore in the sample. Impurity control (e.g., magnetic separation to remove iron particles) is a crucial factor to avoid contaminations, especially in solid-state recycling, where no refinement/melt purification option is possible. In contrast, during remelting, many impurities can be removed, either in the slag or in the gas phase.

### 4.3. Mechanical Properties

#### 4.3.1. Hardness

The microhardness (HV_0.3kg_) measurements for the samples along with their standard deviation, as well as for the reference Al alloys are also presented in Table 3. The measurements were performed after the artificial aging treatment (4 h at 175 °C). A homogeneous microhardness pattern was observed in all samples with low standard deviation values, indicating that the temperature distribution experienced by the samples during sintering was homogeneous. The hardness of the Samples S1–S4 was at the level of 800 MPa, both at the top samples’ top surface and at the cross-sections.

**Table 3:** Hardness measurements of the recycled samples.

Sample	Hardness after artificial aging (MPa)	Standard deviation σ
S1 top surface	780	14.7
S1 cross-section	779	14.7
S2 top surface	777	17.6
S2 cross-section	802	4.9
Al6061-T0 reference	709	20.6
S3 top surface	758	27.5
S3 cross-section	803	36.3
S4 top surface	839	19.6
S4 cross-section	740	39.2
AA6082 T-651 reference	1106	23.5

#### 4.3.2. Elastic Properties

Table 4 presents the results of the IET measurements regarding the elastic properties (E-modulus, G-modulus, Poisson’s ratio, and bulk modulus) of the Samples S2–S4 along with the data for the reference material. The elastic properties of S1 were not measured by IET, due to its shape (not in a disk shape after cross-section). G-modulus can be measured only in the disk shapes (Samples S2–S4). In Table 4, the expected values from the literature [43] for the reference alloys are also presented. Knowing the Poisson’s ratio (*v*) and the Young’s modulus, the bulk modulus (*k*) can be calculated by means of Equation 3:

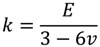
(3)

**Table 4:** Elastic properties measured of the samples and their reference material.

Sample	E-modulus (GPa)	G-modulus (GPa)	Poisson’s ratio	Bulk modulus ** (GPa)
S2 (AA6061 chips)	77.97 ± 0.93	28.98 ± 0.34	0.345	83.84 **
AA6061-T0 reference	69.08 ± 2.48/68–78 *	25–27 *	0.325–0.335 *	65–72 **
S3 (AA6082 chips)	77.34 ± 0.87	28.42 ± 0.32	0.36	92.07 **
S4 (AA6082 chips)	78.95 ± 5.28	29.13 ± 1.95	0.355	90.74 **
AA6082-T651 reference	69.69 ± 0.86/70–74 *	25–27 *	0.325–0.335 *	65–72 **

* Expected values from the literature [43]; and ** calculated from Equation 3.

The elastic properties of specimens were slightly higher, around 10%, compared with the expected and measured values of the parent alloys, as a result of the precipitation and oxide dispersion hardening. The material is stiffer due to the high oxide content (see Table 1), which is dispersed within the material. The results show a linear correlation between the oxide content and the elastic properties; however, the elastic properties in the age-hardening alloys also depend on the heat treatment.

#### 4.3.3. Compression Testing

**Figure 11:**
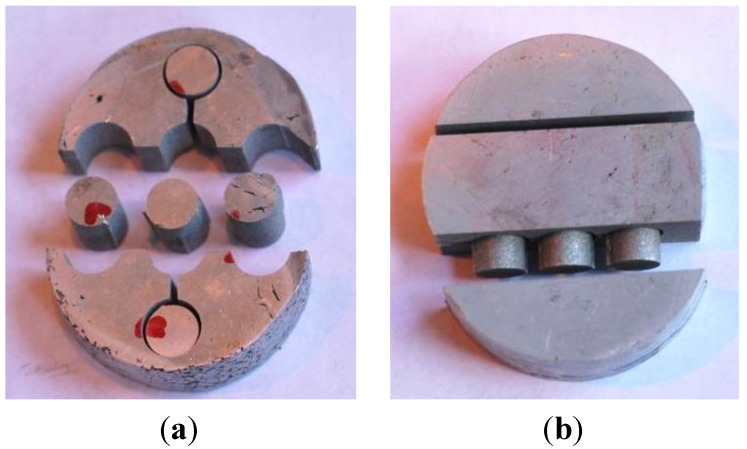
Compression specimens extracted from the SPS samples by electrical discharge machining (EDM): (**a**) five specimens from the top surface of each alloy; and (**b**) three specimens from the cross-section of each alloy.

Compression testing [34] was performed to identify the strain hardening exponent of the SPS samples. To examine the potential influence of the lamellar texture (Section 4.1.2) of the SPS samples in their properties, compression specimens were extracted by EDM, both from the top surface and the cross-section from each alloy. Thus, from each alloy, four compression specimens were taken from the top surface periphery (Top 1–4), one from the top center (top center), and three specimens were taken from the cross-section (Cross 1–2 and cross-center), as can be seen from Figure 11. Moreover, compression specimens were taken from both parent alloys to be used as a reference.

**Figure 12:**
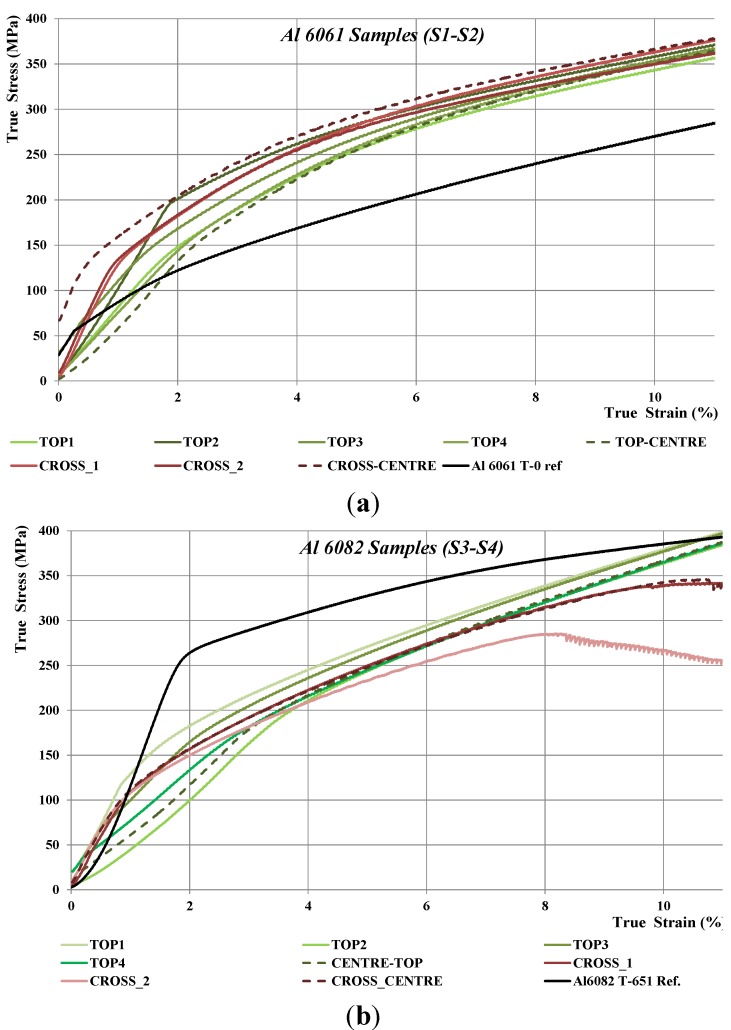
True stress-strain compression curves of the: (**a**) Al 6061 Samples S1 and S2; and (**b**) Al 6082 Samples S2 and S3.

During compression in the plastic region, the compression specimens attained a “barrel” shape. This phenomenon is known as “barreling” and occurs as the frictional force is not constant over the entire cross-section of the specimen and varies from a minimum at the center to a maximum at the edges. The true stress-strain compression curves of the specimens of each alloy taken from the periphery (Top 1–4) and the center of the SPS samples, as well as for the parent alloy used for the chip production as a reference, are shown in Figure 12. The compression specimens from Al6061 (Samples S1 and S2) did not fail during compression testing up to 40% true strain, implying a good ductility and sound welding of powder particles and chips. On the other hand, the specimens taken from the cross-section of Al6082 started to fail at 8% and 11% true strain, as the effect of its lamellar texture and potential void.

For ductile metals, compressive strengths can be derived from the stress-strain curve at specified total strains [21]. The total elongation of the Al6061-T6 is between 12% and 17% [43]. Since necking during tensile loading will occur earlier and the SPS sample is in T5 temper, the compressive strengths are reported at different strains between 8% and 10%.

In the formula σ = *K*ε*^n^*, σ represents the stress on the material, ε is the strain and *K* is the strength coefficient. The value of *n* is between zero, which means that a material is in a perfectly plastic solid, and one, which represents a 100% elastic solid. The strain hardening exponent is obtained as the linear slope of the ln(stress) *versus* ln(strain) curve in the plastic region up to 11% true strain.

The increased strength of the Al 6061 SPS samples compared with the reference Al-6061 T0 is due to the artificial aging step, where the SPS samples gained mechanical properties and can be considered in T5 temper. The Al6082 samples have lower strength compared with the parent alloy used for the chip production. This is due to the fact that the reference material was in T651 temper. Table 5 summarizes the compressive strengths of the SPS samples based on the analysis of true stress-strain curves. Table 5 presents also the strain hardening exponent (*n*), which is a material constant used in calculations for stress-strain behavior in work hardening. The average values of the five specimens along with their standard deviations are shown.

**Table 5:** Compressive strength and strain hardening exponent.

Sample	Strain hardening exponent (*n*)	Compressive strength		
		8% strain	9% strain	10% strain
S1-Al6061 (top)	0.46 ± 0.07	320 ± 5.9	337 ± 6.1	350 ± 5.9
S2-Al6061 (cross-section)	0.40 ± 0.03	336 ± 9.3	348 ± 9.5	360 ± 9.2
Al6061-T0 reference	0.49	240	256	270
S3-Al6082 (top)	0.55 ± 0.05	329 ± 8	351 ± 7	371 ± 6
S4-Al6082 (cross-section)	0.46 ± 0.08	304 ± 16	329 ± 1	342 ± 1
Al6082-T651 reference	0.24	368	376	386

Both alloys had similar behavior and compressive strengths; with the 6082 samples being slightly more ductile compared with the Al 6061 ones, as can been seen from the *n* values. This can be explained mainly due to the fact that all samples had the same heat treatment and process conditions, but the temper of the starting materials were different. Al 6082 chips were in T651 and Al 6061 in T0. Potential “over-aging” of the Al 6082 samples led to a reduction of the strength in favor of improving ductility as a trade-off.

## 5. Conclusions and Discussion

SPS can partially fracture the surface oxides of the Al chips, adsorb the entrapped gases and activate the metallic surface. Summarizing, SPS can be successfully utilized as a novel solid-state scrap welding technique for fabricating fully-dense/void-less billets or near-net or even final shape products, directly starting from Al chips or turnings. The micro-hardness of the bulk recycled specimens is comparable with the cast-based parent material, depending on the heat treatment for the age-hardening alloys. The oxide content of the Al scrap cannot be removed and can act as a hardening factor. Thus, the elastic properties are slightly higher (around 10%) as a result of the oxide dispersion strengthening. The material has a fine equiaxed, grained structure and can be ideally used for applications that require higher stiffness. Apart from the chips’ original micropores, ranging from 5 μm to 20 μm, no significant porosity has been detected, either with microscopy of CT. Only a few micropores were detected in some chip boundaries. CT analysis confirmed the density measurements, showing bulk fully-dense samples. In solid-state processing, there are no refining options compared with conventional recycling, where melt purification is available for many impurities, which can reduce the material properties. Thus, quality control is crucial in solid-state recycling to avoid impurities, such as iron, which usually occur after machining. For example, magnetic separation can easily remove iron particles from the chips. Efficient thermal control of the process resulted in homogenous properties and intermetallic compound distribution.

The Al-Mg-Si system is a precipitation hardening and strengthens from Mg_2_Si. As age hardening alloys, the hardness and strength highly depend on the applied heat treatment. Minor differences in time and temperature during the sintering cycle and quenching can affect the final properties. Thus, optimizing the sintering cycle and quenching (e.g., water quenching), as well as the heat treatment afterwards, the properties can be standardized. Usually, the cycle required to maximize one property is usually different from that required to maximize others. This means that some degree of strength can be sacrificed or “traded off” to improve ductility, as can be seen in the SPS samples, S3 and S4. Generally, the mechanical properties of the SPS samples after the artificial aging step can be considered as comparable with the parent alloy properties in T5 temper.

The authors believe that the mechanisms that result in the efficient surface oxide fracture of the chips and rapid Al scrap consolidation are a combination of spark/plasma generation, plastic deformation between the chips and diffusion bonding, providing additional means in solid-state recycling. In addition, recent studies indicate that the Mg content improves the sintering properties of Al alloys by reducing the oxide layer (with an optimum amount of 0.3–2.5 wt%) [36,37]. Mg is a major alloying element of the 6xxx alloy family from which the chips were generated.

The increase of pressure, sintering time and temperature assist the densification process. However, the process parameters used in this study do not represent the optimum values. Further research will focus on the identification of the optimum parameter set. The overall process time can be significantly reduced by altering certain conditions, such as increasing the heating rate, dwell at elevated temperatures and eliminating Segments 1 and 6 (Figure 3a) by direct quenching of the sample/s in water.

Following the SPS approach described above, Al scrap was consolidated well below the solidus temperature with no physical material losses. Light gauge Al scrap is connected with much higher metal losses during conventional recycling compared with the other scrap categories, making it a relevant feedstock material for solid-state recycling. Insulating the die by graphite paper from the compacted material and the punches forces the current through the compacted chips and, hence, improves the energy efficiency. The FE thermal distribution simulation presented in Figure 3b–e, confirms that SPS is an energy-efficient process, especially if compared to conventional sintering or hot pressing. The homogenous thermal distribution within the material results in homogenous hardness. The FE simulation showed a temperature difference between the measured *TC* temperature and the material temperature, which can be as high as 40 °C during dwelling. Summarizing, the use of SPS as a solid-state recycling technique is technically feasible, highly promising and worth further exploration.

## 6. Future Research

Future research may focus on the optimization of the process parameters, on thermal distribution simulation and on expanding the material scope of this approach. Moreover, the potential environmental benefits achievable by following this recycling approach will be quantified by means of a comparative life cycle assessment study.
